# Pandemic preparedness—political perspectives

**DOI:** 10.1093/sumbio/qvae018

**Published:** 2024-07-13

**Authors:** Harald Brüssow

**Affiliations:** Department of Biosystems, Laboratory of Gene Technology, KU Leuven, Kastelpark Arenberg 21, Leuven 3001, Belgium

**Keywords:** pandemic preparedness, antibiotic resistance, novel antibiotics, non-pharmaceutical interventions, compliance, internet literacy

## Abstract

Pandemic preparedness is explored for the antibiotic resistance crisis and the threat of a next viral pandemic. Bacterial pathogens escaping from control by antibiotics are well defined, and resistance develops over decades while a next viral pandemic occurs suddenly with a novel virus. The death toll for resistant bacterial infections is reviewed, and the scientific and economic hurdles to the development of new antibiotics are discussed. Regulatory adaptations and financial push and pull programs to restimulate new antibiotic development are explored. The COVID-19 pandemic caused not only millions of deaths, but also economic losses in excess of 10 trillion US dollars. Coronaviruses and influenza viruses remain usual suspects for new viral pandemics, followed by paramyxoviruses. Viral infections at the animal–human interface in wet markets and in disturbed environments need active virus surveillance programs. Learning lessons from the COVID-19 for non-pharmaceutical interventions is difficult to draw since measures were frequently applied in combination against different variant viruses and against changing population immunity levels. The Randomised Evaluation of COVID-19 Therapy (RECOVERY) clinical trials demonstrated that even under emergency situations clinical trials can rapidly provide solid treatment data. Various novel vaccine approaches were the most efficient control measures for the COVID-19 pandemic. Pandemic preparedness also requires a fact-based discussion both in the public and in parliaments to settle the conflict between individual freedom and necessary restrictions during a pandemic. Mature and educated citizens are needed not only for coping with pandemics but also for creating stress-resistant democratic societies. Learned scientific societies should contribute to this discussion.

**Sustainability Statement**

The antibiotic resistance crisis and the next viral pandemic represent major infectious disease threats for the 21st century. These two health threats must be addressed within the One Health concept, where human, animal, and environmental health issues are studied in close interconnection. Antibiotic stewardship and novel antibiotic development programs as well as a pandemic treaty need international coordination, substantial financial investments, and political lobbying for public support. The COVID-19 pandemic has revealed substantial discord in the population with respect to public health measures. Microbiologists should become more active in communicating basic knowledge about pathogens and hygiene measures ranging from quarantine to vaccination to foster a rational public discussion on the UN Sustainability Goal 3: Ensure healthy lives and promote well-being for all at all ages.

## What is a pandemic?

Pandemic preparedness requires that one knows what is a pandemic. Literally, this Greek word means all (*pan*) people (*demos*). In the lay press, one speaks of an obesity pandemic, a diabetes pandemic, a fentanyl pandemic, or a viral pandemic. Indeed, lexicographic, scientific, and medical definitions of the word pandemic leave space for interpretation. The Merriam-Webster Dictionary defines a pandemic as “an outbreak of a disease that occurs over a wide geographic area (such as multiple countries or continents) and typically affects a significant proportion of the population.” Epidemiologists concur with this definition (Last 2001). A pandemic thus differs from an epidemic (*epi*, Greek for “near” or “upon”) in its size and geographical spread. The Britannica adds new elements, defining “pandemic is an outbreak of *infectious* disease that occurs over a wide geographical area and that is of high prevalence, generally affecting a significant proportion of the world’s population, usually over the *course of several months* (Pandemic | Description, History, Preparedness, & Facts | Britannica).” The definition of the World Health Organization (WHO) (quoted from "Epidemic, Endemic, Pandemic: What are the differences?" of Columbia University Mailman School of Public Health) adds further elements: a pandemic “cuts across international boundaries, as opposed to regional epidemics or endemic diseases. Based on a disease’s *rate of spread* and its wide geographical reach, pandemics *lead to large-scale social disruption, economic loss, and general hardship*. Pandemic preparedness has thus broad meanings. It could refer to tools to prevent or limit the spread of an infectious disease, but could likewise cover means to alleviate the socioeconomic consequences of a pandemic.

Since definitions of “pandemic” vary, I will in the following focus on two infectious disease conditions that are linked with high mortality and cause high costs: the antibiotic resistance crisis and a viral pandemic, such as COVID-19 or a future viral pandemic. They share a number of characteristics: both threats are contagious, both affect many people all over the globe, and both represent major societal and economical challenges. However, there are also marked differences: the first threat concerns mostly known bacterial infections, while the second represents mainly an unknown viral threat; the antibiotic crisis evolves over decades without a clear end, while a viral pandemic runs over a few years and tends to transform into an endemic viral infection. What unifies both threats is that they represent the two major infectious disease challenges with the potential of disruptive societal effects. Preparedness to counter the effects of both medical crises is thus a societal priority.

The article addresses the UN Sustainability Goal 3: Ensure healthy lives and promote well-being for all at all ages.

## The antibiotic crisis

### Casualties and costs

Infectious diseases represent major health problems. The researchers of the Global Burden of Disease (GBD) consortium estimated 14 million infection-related deaths globally for 2019, making it the second leading cause of death after ischemic heart disease (GBD 2019 Diseases and Injuries Collaborators 2020). Bacterial infections account for about half of these 14 million deaths. Prominent among these bacterial pathogens are *Staphylococcus aureus, Escherichia coli, Streptococcus pneumoniae, Klebsiella pneumoniae*, and *Pseudomonas aeruginosa*. The GBD researchers estimated 1.3 million antimicrobial resistance (AMR)-attributed deaths in 2019 (GBD 2019 Diseases and Injuries Collaborators 2022). Two infectious syndromes dominated the AMR-attributed deaths: lower respiratory tract infections and bloodstream infections. Globally, methicillin-resistant *S. aureus* accounted for more than 100 000 deaths, while Multi Drug Resistant (MDR) tuberculosis, third-generation (3G) cephalosporin-resistant *E. coli*, carbapenem-resistant *Acinetobacter baumannii*, fluoroquinolone-resistant *E. coli*, carbapenem-resistant *K. pneumoniae*, and 3G cephalosporin-resistant *K. pneumoniae* each accounted for more than 50 000 deaths per year.

The GBD estimate for AMR-associated deaths has been confirmed in other studies. The evaluation of the European Antimicrobial Resistance Surveillance Network calculated 670 000 infections with antibiotic-resistant bacteria in 2015 for Europe. These infections resulted in 33 000 AMR attributable deaths, which is close to the 23 000 AMR-associated deaths estimated by GBD for Europe in 2019. Two-thirds of these deaths occurred in health care settings (Cassini et al. 2019). A 2019 report from the Centers for Disease Control and Prevention (CDC) estimated that 2.9 million persons in the USA are infected each year with antibiotic-resistant pathogens, of whom 36 000 die (CDC 2019 report: Antibiotic Resistance Threats in the United States, 2019 (cdc.gov)).

The WHO has declared MDR organisms as a priority area where the development of new antibiotics is urgently needed. The WHO publishes a list of bacteria for which new antibiotics are urgently needed as ESKAPE organisms, which is an acronym comprising the scientific names of six highly virulent and antibiotic-resistant bacterial pathogens, including *Enterococcus faecium, S. aureus, K. pneumoniae, A. baumannii, P. aeruginosa*, and *Enterobacter* spp. The acronym is sometimes extended to ESKAPEE to include *E. coli*.

A frequently quoted projection of the health and economic costs of AMR infections, which was commissioned by the UK Prime Minister (“O'Neil report”), comes to dire conclusions (AMR Review Paper—Tackling a crisis for the health and wealth of nations_1.pdf (amr-review.org)). The projection elaborated by Research and Development (RAND) corporation, a non-profit research organization that develops solutions to public health challenges (About RAND | RAND), and by Klynveld, Peat, Marwick & Goerdeler (KPMG), a multinational professional service network and one of the Big Four accounting organizations (KPMG—Wikipedia) estimated that by 2050, about 10 million people will die per year from infections with AMR pathogens. Asia and Africa will each suffer 4 million AMR-associated deaths; Europe, North America, and South America will see under this scenario each ~300 000 deaths per year. Shocking as these numbers sound, there are also major economic consequences should bacterial infections run out of control. The social costs of such a crisis could cause a reduction of 2%–3.5% in gross domestic product (GDP) by affecting the labor force through mortality and morbidity. It would thus cost the world up to 100 trillion USD over the next three decades. This projection does not yet include social and healthcare costs. Many medical and surgical procedures that are currently conducted under prophylactic protection by antibiotics would become high risk interventions, such as cesarian sections, joint replacements, and chemotherapy, which would translate into a further loss of up to 4% of the GDP. Obviously, the projected GDP loss depends on the model used (The global economic impact of anti-microbial resistance (kpmg.com)). A conservative model has anticipated an absolute increase in current rates of resistance by 40%. Under this model, low-income countries would suffer a 5% GDP loss, while the loss for high-income countries would be 1%. A further model anticipates that, in addition to a 40% increase in the resistance rate, the infection rate by these bacteria might double since antibiotics are less efficient in suppressing the release of pathogens from infected subjects leading to secondary infections. Under this scenario, GDP losses would be 8% and 2% for low- and high-income countries, respectively. Although these projections are widely quoted, researchers have criticized the data because none of these burden estimates themselves have been published in peer-reviewed literature, and it is unclear whether they resist scientific scrutiny (de Kraker et al. 2016).

This uncertainty aside, we can count with between 1 million (calculated in 2019) and 10 million (projected for 2050) yearly deaths due to AMR infections in the future and a partial return to a pre-antibiotic era in medical practice. The O'Neil report concludes that acting quickly is crucial because resistance development is an evolutionary inevitability. Even delaying the resistance process by 10 years could save 65 trillion USD of the world’s economic output between now and 2050.

### New antibiotic development

The GDP study authors recommended five intervention strategies to cope with the problem of AMR-attributed deaths (GBD 2019 Diseases and Injuries Collaborators 2022). Proposed interventions target infection prevention by water, sanitation, and hygiene quality control programs, vaccination for *S. pneumoniae*, reduction of antibiotic use in farming, antibiotic stewardship in human therapy, and investments in the development of new antibiotics.

Sadly, there is a problem with the development of new antibiotics. The golden age of antibiotic development that extended from the 1930s into the late 1950s has long expired. Many antibiotics were discovered by the pharmaceutical industry and introduced into medicine. In the Waksman platform, soil actinomycetes were screened for their ability to produce natural antimicrobial compounds. This approach yielded beta-lactams, aminoglycosides, chloramphenicol, macrolides, tetracyclines, and glycopeptides. Since the 1960s, it became clear that the Waksman platform was overexploited, and it increasingly failed to yield new natural antibiotics (Lewis 2020).

New efforts to develop novel antibiotics were stimulated by progress in bacterial genomics and high-throughput screening (HTS) against chemical libraries (Lewis 2020). At GlaxoSmithKline (GSK), many bacterial target proteins were screened against up to half a million chemical compounds. However, the level of success was unsustainably low in comparison with screening outcomes for other therapeutical areas. Similar experiences were made by many other companies (Chan et al. 2004). AstraZeneca reported objective technical hurdles to identify promising lead compounds by HTS that they attributed to problems of drug permeation into bacterial cells (Tommasi et al. 2015). Subsequently, major pharmaceutical companies have shifted their commercial focus away from antibiotic development. While venture investment for cancer drugs has steadily increased over the last decade to US$7 billion, investments in antibiotics over the same time period remained flat and did not even reach a quarter billion US$ in 2020 (McKenna 2024).

This leads to a paradoxical situation: When antibiotic resistance reached critical levels, the pharmaceutical industry abandoned programs to develop new antibiotics. This industrial decision seems counterintuitive, but is understandable when considering scientific and economic hurdles to develop new antibiotics.

### Scientific hurdles

Let us first consider one of the technical hurdles. Gram-negative bacteria are surrounded by two membrane systems. This dual membrane system represents a formidable barrier for drug penetration into bacterial cells that frustrated screening programs in pharmaceutical industry and represented a nearly insurmountable challenge for medicinal chemists to design synthetic antibiotics.

Encouragingly, substantial scientific progress has been achieved over recent years to define the chemical properties needed to overcome the permeation barrier in Gram-negative pathogens; by extending the chemical space of antibiotic candidates; by full modular synthesis of suitable molecules; by bioprospecting of previously “unculturable” bacteria or unusual bacteria; by attacking bacterial targets on the outer bacterial membrane; and by looking for support from structural biology, genomics, molecular genetics, and phylogenetic analyses to promising candidate antibiotic discovery (for a recent review, see Brüssow 2024a and Ardal et al., 2020). This is an active research area with numerous recent additions ranging from deep learning approaches screening metagenomes and prokaryotic genomes thus identifying nearly 1 million antimicrobial peptides; 79 from 100 peptides synthesized and tested showed activity against pathogens (Santos-Junior et al. 2024), to the design of antibiotics targeting the lipoprotein transport system of Gram-negative bacteria that spares the gut microbiome in mice preventing secondary infections with *Clostridioides difficile* (Munoz et al. 2024). However, these research activities were mostly conducted by academic researchers and small biotech companies. This research identified antibiotic candidates, elucidated their mechanism of action, and demonstrated their *in vivo* efficacy in standard mouse infection models, but this research has stopped at this level. Due to limited financial resources and academic orientation, universities shied away from conducting clinical trials.

### Economic hurdles

To understand why the pharmaceutical industry has left the field of novel antibiotic development, let us first consider the cost of developing a new antibiotic. Two reviews assessed research and development expenditure on new therapeutic agents approved by the US Food and Drug Administration (FDA) between 2009 and 2018 (Wouters et al. 2020, Schlander et al. 2021). Anti-infective agents for systemic use had a mean development cost of 1.3 billion USD, which is close to the average cost of getting a drug to the market, namely 1 billion USD. In comparison, the development costs of anti-neoplastic agents were 2.7–4.5 billion USD. This price calculation includes the cost of failed drug trials. The price tag associated with the development of antibiotics is thus not a factor that has discouraged the pharmaceutical industry.

However, the attrition rate for anti-infectious agents in clinical trials has increased. Antibacterial drug development programs initiated in the 1980s and 1990s obtained marketing approval in 40% of cases. For drug development programs initiated between 2000 and 2009, the approval rates had decreased to 23% (Dheman et al. 2021). Even more discouraging are peculiar characteristics of the antibiotic market with respect to the prescription practice of antibiotics, which discourage financial investments into the field. First, resistance to new antibiotics tends to develop relatively early after the introduction of a new antibiotic and increases in prevalence with time, thus limiting the commercial time window for a return of investment. Second, prescription practices, such as the WHO-recommended and from a public health viewpoint reasonable “AWaRe” strategy (Hsia et al. 2019, Zanichelli et al. 2023), make it uncertain whether a new valuable antibiotic will become a commercial success. “A” stands for “Access” and describes antibiotics recommended as first- or second-line therapies for specific infectious diseases. “Wa” stands for “Watch,” a stewardship program for antibiotics sensitive to induce resistance development. “Re” stands for “Reserve” and describes last-line antibiotics only to be used in life-threatening infections with AMR pathogens. While this approach will prolong the longevity of some last-line antibiotics, it curtails the revenue of pharmaceutical industries when putting valuable, novel antibiotics on the market. As a consequence, only 43 phase 1–3 clinical trials were registered for antibiotics at the end of 2020 compared to 1300 trials for anticancer agents.

### Regulatory changes

Traditional market forces are thus unlikely to provide a solution to the antibiotic crisis. Pandemic preparedness therefore calls for supportive measures to remotivate industry to invest in the development of novel antibiotics. Since the clinical trials represent the majority of the development costs and time, changes in the approval process might be needed. Indeed, the FDA implemented the Fast-Track Program in 1987 and the Accelerated Approval Program in 1992, which shortened the review time from 3 years to <1 year. In addition, new FDA approvals supported by at least two pivotal phase 3 clinical trials decreased from 81% to 53% over the last 30 years. However, review fees increased from 66 million to 820 million USD (Darrow et al. 2020). The European Medicines Agency (EMA) has been issuing conditional marketing authorizations since 2006. These changes imply a shift in R&D costs from pre- to post-marketing authorization. At the FDA, an expedited drug development approach is under discussion that would lead to restricted use of the product after approval. So-called limited population antibacterial drugs are considered for the treatment of MDR microbial infections after a very limited clinical development program, based on many *in vitro* and *in vivo* modeling studies for a special approval. The stakeholders are exploring efficient ways of fostering collaboration on post-authorization data collection that can then meet full regulatory needs (Baird et al. 2014). Clinical trials are major cost factors for drug development: Preclinical development times for antibiotics are of 4–5 years, while clinical development times exceeds now 8 years (Outterson and Rex 2023). To reduce the cost of launching novel antibiotics, it was also proposed to replace cost-prohibitive large randomized, double-blind clinical trials by adaptive licensing strategies as for orphan diseases where a restricted approval is given for specific infections with MDR pathogens (Cook and Wright 2022).

### Financial push programs

Research and early development of novel antibiotics are now also receiving direct financial support. Combating Antibiotic-Resistant Bacteria Biopharmaceutical Accelerator (CARB-X) is a global non-profit partnership accelerating antibacterial products to fight drug-resistant bacteria (Wayback Machine (archive.org)). CARB-X, founded at Boston University, funds the early development pipeline of new antibiotics, vaccines, and rapid diagnostics with money from governments (the USA, Germany, the UK, and Canada) and foundations. In its first funding period (2016–21), it has supported 92 projects with 361 million USD. Projects included the development of a new inhibitor against carbapenem-resistant Enterobacteriaceae by Entasis Therapeutics; the development of a microbiome consortium against AMR bacteria by Seres Therapeutics; first-in-human programs with a topoisomerase inhibitor (by Bugworks); a drug against an adhesion protein from *E. coli* (by GSK); antimicrobial peptides (by MicuRx); and a monoclonal antibody (by Trellis). A portfolio against drug-resistant *Neisseria gonorrhea* was funded as well as therapeutic bacteriophage development and drugs targeting bacterial virulence factors. In 2022, the NIH, Wellcome, and several governments committed renewed funding with an additional US$370 million to CARB-X.

Further push programs for novel antibiotic development were created. Established by the WHO in 2019, Global Antibiotic Research and Development Partnership (GARDP) is a not-for-profit research and development organization, with a focus on late-stage clinical development (Balasegaram and Piddock 2020). With a budget of 90 million euros, its targets are sexually transmitted infections [where it has jointly funded with Entasis a phase 3 clinical trial of zoliflodacin against *N. gonorrhea* (Taylor et al. 2018), neonatal sepsis, and infections in hospitalized adults and children]. GARP spends now more than 100 million euros per year on its projects (GARDP Strategy 2020–2025: Uniting Against Antibiotic Resistance | GARDP). Another initiative is Replenishing and Enabling the Pipeline for Anti-Infective Resistance, funded by the Novo Holdings Initiative with 165 million USD, which sponsors compounds that are between lead optimization and phase I clinical trial, a critical phase in antibiotic development with high failure rates (Engel 2020).

An analysis of the situation by the initiator of the CARB-X initiative concluded that some progress has been achieved with these push programs, but the “10 × 20” call of the Infectious Diseases Society of America from 2002 to introduce 10 new antibacterial antibiotics by 2020 has not been achieved (Rex and Outterson 2021). An analysis of investigational new drug applications (INDs) for new, systemic antibacterial drugs between 1980 and 2019 showed unsettling trends (Dheman et al. 2021). A peak was reached in 1987 with 39 INDs. This declined to 13 INDs in 2001; this number recovered to 34 in 2009, followed by a slow decrease thereafter. More important is the shift in companies having antibiotics in clinical development: while the majority were large pharmaceutical industries until 2001, this changed thereafter with biotech companies taking the lead. In 2020, only three large US pharmaceutical companies remained in the antibiotic field with clinical development. There was another important change: The success rate of INDs advancing to marketing approval was >40% after 6 years of review during the 1980s and 1990s, while thereafter this rate fell to 23% after 8 years of review. In 2019, the majority of INDs came from already established drug classes. The US Generating Antibiotic Incentives Now Act from 2012, which made products eligible for priority FDA review and 5 years of additional marketing exclusivity, did not reverse the trend. Financial figures are also not favorable: initial sales routinely achieve <$100 million/year for new antibacterial agents despite much higher developing costs. Under these conditions, biotech companies went rapidly bankrupt (Tetraphase sold for $14 m … and $600 m goes up in smoke! • AMR.Solutions).

### Financial pull programs

Rex and Outterson (2021) argued that, in addition to well-intentioned push mechanisms, pull efforts are also needed to revive the antibiotic pipeline to a level needed to affront the ongoing antibiotic crisis. The reduced success rate of antibiotic clinical development reflects improved clinical trial quality, reflecting increased scientific knowledge, but this improved quality further discouraged investments in the field. To compensate for these difficulties, the authors concluded that it needs post-approval “pull” incentives. This could be achieved via a restructured reimbursement in a volume-independent fashion. As in the COVID-19 pandemic, where governments bought large amounts of vaccine in advance, governments could buy access to new antibacterial agents and justify this as a measure of pandemic preparedness.

The pioneer in the pull activity is the UK government with its National Institute for Health and Care Excellence (NICE) program. The pull incentive is a reimbursement for new antibacterials that is delinked from volume of sales. In this way, the development of new antibiotics could be stimulated while maintaining an antibiotic stewardship. The first two agents have been selected and are supported with £10 million per year per agent. Outterson and Rex (2023) acknowledge that this initiative could become a game changer in an economic situation where the market forces are unable to assure innovation and new products, but criticize that the choice following strict health technology assessment criteria is rather conservative since NICE supported agents that are related to an existing class of beta-lactams and thus does not provide much novelty. Furthermore, higher performing drugs should receive a higher reward in the future than the capped £10 million support per year. Finally, the UK is a too small market for solving the problem of access to badly needed novel antibiotics. Similar programs should be established by other rich countries, which should consider these investments as a type of health insurance for their citizens in the future. International cooperation is needed since several billion USD of support are needed to realize a potent pull factor and because the antibiotic crisis concerns all countries. AMR pathogens do not know frontiers in a globalized and highly connected world.

The PASTEUR Act (The Pioneering Antimicrobial Subscriptions To End Up Surging Resistance Act) first planned with 11 billion and then cut to 6 billion USD would provide the critical mass of money to establish a subscription-style model where the US government enters into contracts with antibiotic developers, paying upfront for access. Under this bill, antibiotics would be sold based on their value to public health rather than via volume sales to recoup development costs [Fighting AMR with a value and subscription model—Pharmaceutical Technology (pharmaceutical-technology.com)]. Sadly, the PASTEUR Act twice did not pass the US Congress (McKenna 2024).

### Alternatives to antibiotics?

Other potential treatment modes of antibiotic-resistant bacterial infections are gaining attention in the scientific community. One of them is bacteriophage therapy, which has a colorful history in the Soviet Union where it served for decades as an alternative to antibiotics. The outcome of larger controlled randomized phase II clinical trials has so far not been encouraging, raising questions about the use of preformulated bacteriophage cocktails (Sarker et al. 2016, Leitner et al. 2021). More promising were case studies of individual patients treated with personalized phage preparations (Uyttebroek et al. 2022). A cumulative evaluation of 100 consecutive patients suffering from respiratory, skin, soft tissue, and bone infections with personalized phages by Belgian researchers showed clinical improvement in 77% and pathogen eradication in 61% of the cases. Eradication was 70% less probable when no concomitant antibiotics were used suggesting phages as an adjunct but not as an alternative to antibiotics (Pirnay et al. 2024).

Drawing conclusions from the published literature on the use of probiotics as antimicrobial agents is difficult since rarely more than two clinical trials were conducted with the same probiotic strain against the same medical condition (for a review, see Brüssow 2019). The European Society for Paediatric Gastroenterology, Hepatology and Nutrition made a few recommendations for probiotic use against acute gastroenteritis and antibiotic-associated diarrhea. It formulated a conditional recommendation with low certainty of evidence to provide either *Lactobacillus rhamnosus* or a combination of *Bifidobacterium* and *Streptococcus thermophilus* probiotics to reduce necrotizing enterocolitis rates in preterm infants (van den Akker et al. 2020).

## Viral pandemics

### Influenza and coronaviruses

Compared to the antibiotic crisis, viral infections represent distinct challenges with respect to pandemic preparedness. When considering viral pandemics over the last century, infections with three virus groups stick out: influenza virus causing the Spanish flu pandemic (Brüssow 2022a ) and several smaller pandemics; the lentivirus, HIV, causing the AIDS pandemic (Scully and Kuritzkes 2023); and coronaviruses causing the COVID-19 pandemic (Beigel and Uyeki 2023) and several smaller epidemics (MERS, SARS) (Perlman and Masters 2021). Compared to the antibiotic resistance crisis, one major difference of viral pandemics is their onset speed. The time interval from the first SARS-CoV-2 cases to a local epidemic in Wuhan and the spread of the epidemic over all of China and then followed by a worldwide spread was a matter of a few months. This speed does not leave much time for setting up containment measures. However, early warning signs existed. Influenza viruses cause regular seasonal epidemics and are notorious for avian and porcine influenza virus epidemics that occasionally spillover into substantial numbers of human subjects (Brüssow 2024b ). Coronaviruses are also well known for trans-species infections and crossed repetitively into humans in recent decades, most notably in the smaller SARS pandemic from 2003. There is even circumstantial indication that the Russian flu pandemic from 1890 might have been a prior coronavirus pandemic (Brüssow and Brüssow 2021). Based on this previous pandemic experience, influenza viruses and coronaviruses are strong candidates for a further viral pandemic.

### Zoonosis and Nipah virus

In addition, numerous epidemics with other viruses have been documented in recent decades underlining that one cannot restrict surveillance programs to these two viral groups. Climate change, humans encroaching increasingly the environment of wild animals, global connection by tourism, and trade are all factors that increase the likelihood that humans will be exposed to new viral infections. Ecologists predict that global warming will cause animal migration to higher latitudes and higher altitudes to escape heat spells. This will lead to an exchange of viruses between animal species that have not met in the past (Carlson et al. 2022). One might suspect that some of these novel cross-species infections will preadapt viruses for a further spillover into the human population. Also, the expansion of agricultural areas into the tropical rainforests exposes humans to viruses from animals that have lost their natural habitats. The classical case is Nipah virus infections, a paramyxovirus causing severe pneumonia (Lee et al. 2021). The clearing of tropic forests for oil palm tree plantations in Malaysia forced starving fruit bats into farms practicing combined pig husbandry and mango cultivation. Fruits contaminated with the saliva from bats dropped to the ground, where pigs ate the fruits and contracted a respiratory infection. Pigs served as an intermediate host that then transmitted the infection to farmers and slaughterhouse workers. Even more scary is the observation that in India and Bangladesh, Nipah virus from fruit bats could infect humans without an intermediate animal host via direct contamination of food (palm sap drinks). In addition, upon close contact with patients, human-to-human transmission of highly lethal Nipah virus infections was observed. Nipah virus might therefore represent a further future pandemic threat.

### Wet markets

Close encounters of humans with wild animals represent a risk situation for cross-infections of humans with animal viruses. Virologists have long suspected bushmeat consumption in tropical Africa as source for some newly emerging infections. Close encounters with animal viruses also occur in traditional wet food markets, creating a hot spot for viral spillover events when live wild animals are sold as culinary delicacies. Several cross-infections with avian influenza virus have been traced to live poultry markets. Behind such markets are wildlife animal farms and illegal trafficking of wild animals (e.g. pangolins) that create animal–human interfaces for the exchange of viruses. Although the origin of SARS-CoV-2 has not been defined, it is notable that all early COVID-19 cases in Wuhan clustered around one particular wet market in the city.

Pandemic preparedness thus starts with a surveillance of critical animal–human interfaces where virus spillover can occur and practices that minimize spillover risks. A prime target here is wet markets. Closing wet markets is probably not a practical idea since the livelihood and food for too many people depend on them and such measures would meet fierce opposition from the population. However, health and food authorities could introduce measures that prevent the mixing of different animal species and a too close contact of animals with food handlers and clients combined with appropriate hygiene measures and surveillance. One might, however, ban the selling of wild animals because the greatest virological risk is associated with their handling. Skeptics argue that forbidding the selling of highly valued wild animals as food or folk medicine would only lead to an uncontrollable underground market. A regular control of the animals and food handlers on wet markets for viral infections could represent valuable sentinel studies allowing early warning of pending spillover infections (for a recent review, see Brüssow 2023).

### Historic precedence

Exchanges of viruses between animals and humans are not new phenomena. They probably occurred in the distant past since the time when humans domesticated animals. The close genetic relatedness between the rinderpest virus and the human measles virus is likely a testimony of such an early viral cross-species infection event between bovines and humans. Historians and virologists have proposed that smallpox infections were introduced into the human population with the domestication of camels in Arabia and its introduction as transport animals in Ancient Egypt, where mummies with pock scars were identified. When these viruses were imported by the Spanish conquerors into the New World where cows and Old World camels were unknown, a dramatic collapse of the indigenous population was the consequence, demonstrating the threat of new viruses meeting a susceptible, immunologically naïve population (Brüssow 2023).

### Viral surveillance programs

Worldwide surveillance systems exist already that study currently circulating influenza viruses. These data are used to adapt the annual seasonal influenza virus vaccines to the actual prevailing epidemiological situation. The COVID-19 pandemic has stimulated surveillance activities to detect new SARS-CoV-2 variants circulating in the human population. Such programs are valuable early warning system for emerging SARS-CoV-2 variants that are not covered by the immunity induced by current vaccines. Combined with artificial intelligence approaches, the bioinformatic analysis of SARS-CoV-2 genome sequences has led to the EVEscape program. When trained with coronavirus genome data from the pre-pandemic period, EVEscape was able to predict future viral variants that fitted variants later observed during the COVID-19 pandemic. Such predictions could allow the preparation of correspondingly modified mRNA vaccines (Thadani et al. 2023).

Critical animal–human interfaces, created, e.g. by logging or agricultural activities in recently cleared tropic forests, should also be targeted with surveillance efforts. The detection of viral genome sequences in wastewater and sewage offers here a powerful new tool of surveillance (Karthikeyan et al. 2022). Pandemic preparedness might also call for a more targeted virological analysis of febrile illnesses in people working at these interfaces. After exclusion of common causes for febrile illness, blood samples could be investigated by metagenome sequencing or viral isolation. Such approaches worked successfully in Chinese febrile patients with unknown etiology, which led to the identification of a novel paramyxovirus, the Langya virus (Zhang et al. 2022). The Chinese study is not an exception: similar approaches have led to the detection of a porcine delta coronavirus in children from Haiti with fever, of a canine coronavirus in children from Malaysia, or of a rat arenavirus in children from Cambodia (for a recent review, see Brüssow 2024c).

Surveillance efforts should not be limited to human cases. It is undisputed that pandemic preparedness builds on the One Health concept, where human, animal, and environmental health are intricately connected. Veterinary surveys studying the dynamics of H5Nx high-pathogenicity avian influenza virus epizootics demonstrated that they had their origin in Asia and subsequently spread over wide geographical areas and from domesticated birds to wild birds (for a recent review, see Brüssow 2024b). Such a spread increases the probability of humans being exposed to animal viruses under conducive ecological conditions allowing a viral spillover. Indeed, H5N1 avian viruses have already infected marine mammals in South America. Cattle and at least two farmers in the USA have been infected with avian influenza virus (Cohen 2024a ).

Some virologists argue that pandemic preparedness also requires a better knowledge of viruses infecting game animals that are appreciated as food delicacy (e.g. racoon dogs, civets) or as folk medicine (e.g. pangolins), particularly in China (He et al. 2022). These animals come in close contact with humans via trade, hunting, and wildlife farming. A focus on viruses from these animals is certainly justified since they were discussed as possible intermediate hosts in the COVID-19 and SARS pandemics. Other virologists suggest that systematic surveys should investigate virus carriage in bats, rodents, and shrews since bats and rodents are frequently discussed as potential sources for zoonotic infections (Chen et al. 2023). Their worldwide distribution, high species numbers, large population sizes, and wide action radius (flight in bats) made them common suspects when discussing the origin of pandemics. Still, other virologists suggested to extend sequencing to characterize the virome of all mammalian species. The United States Agency for International Development (USAID) launched such a project in 2021. Since it included virus isolation, the project was canceled in 2023 because the risk of propagating viruses with pandemic potential was considered as too high. Researchers voiced their disagreement with this decision and argued that a better knowledge on animal viruses is a cornerstone of pandemic preparedness. The USAID decision responded to political pressure sparked by discussions whether SARS-CoV-2 originated by an escape of a bat virus isolate at the Wuhan Institute of Virology. The origin of SARS-CoV-2 is still unclear, and pandemic preparedness needs a rational discussion of biohazard risks in working with potentially dangerous viruses. Pandemic preparedness needs in any case transparency, openness, and the sharing of information during the initial phase of any outbreak of a novel infectious disease, which was missing during the initial phase of the COVID-19 pandemic.

### Non-pharmaceutical intervention learning lessons

Pandemic preparedness depends a lot on learning lessons from the COVID-19 pandemic. Public health authorities in Germany did for example an excellent job when tracing the infection chain from an infected Chinese individual traveling to a conference in Munich in the very early phase of the pandemic (Böhmer et al. 2020). However, when the case numbers increased, test results were communicated via FAX machines from local public health offices, demonstrating that information technologies were not yet introduced and slowed communication in the German public health sector. An app for tracing contacts on smartphones was later introduced in several countries, but privacy issues delayed its introduction. Pandemic preparedness requires that such measures must be developed proactively, that technical, political, and legal issues must be discussed and settled before rather than during a pandemic, and public health employees must be trained in prior drill exercises. There were other indications of apparent pandemic unpreparedness (Timmis and Brüssow 2020): e.g. medical doctors lacked trivial items such as face masks in the initial phase of the pandemic. It seems obvious that personal protection items, at least for the health sector, should be stored at local civil protection centers or centrally at the army. Additionally, countries famous for their research and public health institutions, as well as regulatory authorities initially revealed shortcomings with the introduction of diagnostic tests. A critical failure and gap analysis of such shortcomings are needed, and correcting actions should be part of future pandemic preparedness programs.

Taking learning lessons from non-pharmaceutical intervention (NPI) measures to curb the spread of COVID-19 is not easy (for a recent overview, see Brüssow 2024c). It might be understandable that in an emergency situation political authorities imposed several control measures simultaneously. However, this makes it very difficult to differentiate the effect of individual measures. Effects were deduced from slightly staggered introduction of different control measures, but the statistical power to differentiate effects of single measures was small. An efficiency analysis is further complicated by the dynamic nature of the COVID-19 pandemic, which consisted of waves with distinct variant viruses differing in virulence and transmissibility such that observations from one wave were not easily transferable to another infection wave. Further complicating factors for a straightforward analysis of the efficacy of non-pharmaceutical measures was the dynamic nature of the immunity in the population acquired by natural exposure or vaccination.

Controlled randomized trials for individual NPI tools during the COVID-19 pandemic are mostly lacking. Admittedly, such trials are difficult to conduct, impossible to blind, and hard to organize or justify politically during an emergency situation. For example, with respect to face mask wearing a decently powered intervention trial was only conducted in Bangladesh (Abaluck et al. 2022). The remainder of the evidence comes from a few underpowered trials and observational studies. An interesting early study compared the introduction of mandatory face masks in the German city of Jena with neighboring cities where no mandates were issued (Mitze et al. 2020). Suggestive evidence for a protective effect of face mask wearing was deduced from this comparison. One would wish that carefully controlled trials for NPI measures would have been organized in matched regions applying or not a single NPI measure, followed by an evaluation when sufficient numbers of infections are reached to assure statistically significant conclusions. Many NPI measures, including face mask wearing, were controversially discussed in the public, and opinions were strongly influenced by politically motivated prejudges. Hard data might have led to a more rational approach to the value of NPIs and greater acceptance of restrictions that had proven their efficacy. Due to strategies taken by governments of different countries or during different pandemic phases in a single country, some evidence exists for the efficacy of different forms of lockdowns and curfews (for more references, see Brüssow 2024c). None of these conclusions was based on the comparison of deliberately planned trials in neighboring cities. Only with the help of sophisticated statistical analyses, it was possible to conclude on the efficacy of individual NPI measures when several NPI measures were generally introduced concomitantly.

### The importance of controlled trials

It might sound a bit naive to plan and conduct such trials under conditions of a pandemic. However, outside of a pandemic situation, such data cannot be obtained, and the medical sector has demonstrated that controlled clinical trials can be conducted under a pandemic situation. In fact, the UK National Health System achieved to conduct the RECOVERY clinical trial to evaluate the efficacy of treatment modes for COVID-19 patients. The RECOVERY trial enrolled most patients hospitalized with COVID-19 pneumonia in the UK. It was a randomized, controlled, open-label trial. RECOVERY is a multi-arm adaptive clinical trial that was quickly set up such that results were obtained rapidly. Inefficient treatment modes, once demonstrated, were replaced by new treatment modes in the test (the RECOVERY trial is 2 years old today—RECOVERY trial). Thanks to the centralized British NHS structure, the results became available during the pandemic, were approved, and could allow an evidence-based clinical treatment of severe COVID-19 pneumonia. This situation contrasts well with the widespread treatment of COVID-19 patients with hydroxychloroquine, which was based on very weak and unconvincing *in vitro* experiments, much hype, and no scientific evidence but experienced nevertheless prominent political support. The uncritical acceptance of an inefficient treatment (RECOVERY proved the inefficacy of hydroxychloroquine) has probably caused more harm than waiting for the results of clinical trials (RECOVERY proved the efficacy of low-dose dexamethasone).

It is true that the RECOVERY trial might have been difficult to conduct outside of the UK NHS system due to different organizations of health care in other countries. However, a pandemic is a planetary emergency, and it is sufficient that critical trials are conducted in countries that offer the best conditions to achieve results in a short time. Transparency and international collaboration are crucial during a pandemic. Coordination by a central organ such as WHO is important.

### Vaccination

Vaccines against SARS-CoV-2 were developed with warp speed. A multitude of vaccine types were explored by universities, biotech companies, and big pharma supported by governmental push and pull financial programs. The efficacy of several vaccines was clearly demonstrated in impressive series of carefully conducted clinical trials and approved for mass vaccination. Approved vaccines comprised entirely new types such as mRNA vaccines (for a review, see Brüssow 2022b). The vaccines were game-changers during the pandemic despite some shortcomings. Shortcomings were political and technical. Politically, existing efforts must be intensified to assure an equilibrated distribution of vaccines to low-income countries that could not afford to pay market prices for commercially developed vaccines. Technically, the need for cold chains and the logistic problems linked to repeat immunizations in the distribution of mRNA vaccines calls for more scientific efforts to develop vaccines suitable for low-income countries. In addition to these political and economic problems, injected vaccines do not induce a mucosal immune response and had thus only a moderate effect on virus excretion in vaccinees that were still experiencing breakthrough infections. However, the approved vaccines protected efficiently against severe disease and death. The immune response waned with time, necessitating booster injections to maintain immunity. The evolution of a series of viral variants diminished the neutralization of variant viruses by antibodies induced by vaccination, but again booster immunizations were a partial remedy. In the meanwhile, researchers and industry developed vaccines against multiple and variant viruses, as well as vaccine forms that induced protective mucosal immunity that could interfere with the transmission of infections (Ye et al. 2023). Overall, this surge of vaccine research and development if maintained will probably be our best bulwark against the next viral pandemic should it be a respiratory disease. It might be desirable to extend these novel vaccine technologies also to other respiratory viral groups. The paramyxovirus respiratory syncytial virus might be a suitable, commercially attractive field providing insights that might be essential if viruses such as Nipah virus learn to efficiently spread in the human population.

Antivirals were not a similar success story. RECOVERY clinical trials demonstrated that a cocktail of two anti-viral monoclonal antibodies, but not convalescent plasma, showed efficacy in COVID-19 patients. In contrast, the chemical antivirals Lopinavir/Ritonavir, an HIV drug, lacked efficacy in COVID-19 patients. Clearly, it will be important to also extend antiviral research as an investment into pandemic preparedness (for more references, see Brüssow 2021).

## The economic costs of pandemic preparedness

Pandemic preparedness goes beyond scientific research trying to find new antibiotics against resistant bacteria and early detection of new viral pandemic threats. Pandemic preparedness is a task for our societies. The global nature of a pandemic calls for solutions at a global level.

The WHO is currently holding its 77th World Assembly in Geneva, and it is not a promising sign that the member states cannot (at least at the time of the writing) agree on a pandemic treaty to be better coordinated in the case of a next pandemic (Cohen 2024b). Contentious points brought forward by African governments are the rapid sharing of clinical samples containing novel pathogens from new outbreaks, the transfer of technology, the patent lifting for vaccines and drugs, and their fair sharing and distribution during a pandemic. African governments also object to treat pandemic threats within the framework of the One Health concept. WHO, as a United Nations organization, is, in principle, the ideal coordinator during a pandemic, and it is hoped that a compromise for a pandemic treaty can still be found.

Rapid sharing of new data on emerging infections is a crucial aspect of such a pandemic treaty. Clinical and epidemiological details on new infections should be documented, ideally in a centrally maintained data repository run by the WHO. Clinical samples should likewise be sent to regional and specialized virus reference centers, allowing a speedy diagnosis by sequencing analysis and if possible by virus isolation. As critical infections might emerge in low-income countries, financial support of regional diagnostic centers by high-income countries will be needed. Low-income countries might correctly complain that in return for providing critical clinical samples, they later need a fair share to diagnostic tools and vaccines developed on the basis of this shared clinical material. Pandemics are best addressed by global containment efforts. While politically understandable, limiting diagnostics and vaccines to high-income countries that developed these tools is not only a selfish reaction, but in view of the global spread of infections an irrational and counterproductive approach.

The organization of control measures during a pandemic requires substantial financial resources. During the COVID-19 pandemic, the WHO had an annual budget of about US$6 billion (4.4 billion base budget, 0.5 billion polio eradication, 0.2 billion special programs, and 1 billion US$ emergency operations) (proposed program budget 2022–3 (who.int)). The greatest share is volunteer contributions (major contributors are the US with 1 billion, Germany with 0.6 billion, Gates Foundation with 0.8 billion, Gavi with 0.5 billion, and the EU with 0.4 billion US$) (*Le Temps* (Switzerland newspaper) from 28th May 2024). If one compares this WHO budget with that of the University Hospital of Geneva, which amounts to about US$2 billion per year, serving a single Swiss canton, it becomes clear that the WHO is insufficiently financed by its member states to assure global health and pandemic preparedness.

It is revealing to quote from a report of the Global Preparedness Monitoring Board (GPMB), issued in September 2019, just ahead of the COVID-19 pandemic (A_world_at_risk.pdf (mm8.dk)). “For too long, we have allowed a cycle of panic and neglect when it comes to pandemics: we ramp up efforts when there is a serious threat, then quickly forget about them when the threat subsides. It is well past time to act.” It is an unfortunate conclusion that after a pandemic is before a pandemic. Complacency is not in place. The report claims that “Heads of government must commit and invest.” To better motivate the taxpayer for investments, “Financing institutions must link preparedness with financial risk planning.” It will then become apparent that billions of investments in pandemic preparedness are small in comparison to the trillions of economic damage caused by a pandemic. In October 2020, a report in a medical journal calculated the cost of the COVID-19 pandemic to 16 trillion US$ (Cutler et al. 2020). The World Economic Forum (WEF) calculated US$ 11 trillion spent on the COVID-19 response up to October 2020. At the same time, the WEF estimated that it would have cost 50 billion US$ to prepare the world for a pandemic [Report: COVID-19 has cost $11 trillion so far, but it could have been avoided | World Economic Forum (weforum.org)]. Even when considering only the first months of the COVID-19 pandemic, pandemic preparedness would achieve a substantial return on investment.

The GPMB continues that “The world is confronted by increasing infectious disease outbreaks” and continues that “All economies are vulnerable, while the poor suffer the most.” In 2019, they concluded that “The world is not prepared for a fast-moving, virulent respiratory pathogen pandemic.” Sadly, the COVID-19 pandemic confirmed the validity of their assessment.

## Political aspects of pandemic preparedness

GMPB raised in September 2019 another important point, namely “Trust in institutions is eroding. Governments, scientists, the media, public health, health systems and health workers in many countries are facing a breakdown in public trust that is threatening their ability to function effectively. The situation is exacerbated by misinformation that can hinder disease control communicated quickly and widely via social media.” This erosion process accelerated during the COVID-19 pandemic. The human cost of the COVID-19 pandemic was high: an estimated 15 million deaths occurred worldwide, and in a survey, 6% of the British population suffered from COVID-19 symptoms for at least 3 months, which would worldwide translate to many millions of long COVID cases (Else 2022). Likewise, the economic costs were towering. The Economist calculated that the pandemic could amount to USD 10 trillion in forgone GDP over 2020–21 alone (What is the economic cost of COVID-19? (economist.com)).

However, the most serious and enduring effects of the pandemic might be on a societal and political level. Historians had speculated that the Russian flu pandemic of 1890 might have contributed to the “Fin de siècle” feeling that led to the destabilization of the European order at the beginning of the 20th century. It might not be farfetched to suspect that the COVID-19 pandemic and the reaction of part of the population against governmental actions to control the spread of the pandemic provided a stimulus to populist movements, further eroding trust in governmental institutions. Pandemic preparedness should thus also encompass substantial efforts to counteract misinformation and deliberate disinformation. A GMPB conclusion drawn before the COVID-19 pandemic thus remains valid “Social science research is poorly integrated into national and international research portfolios, and not applied to preparedness.”

Trust is rapidly lost, but difficult to gain. Transparency in political decision-making is an important element of handling a health crisis such as a pandemic. Clear communication also on topics that cannot yet be settled on the basis of available evidence is crucial. Politicians need to engage citizen with their decisions, imperfect as they might be at the moment. Media also have a duty, which is not limited to provide correct information. “We provide learning resources to empower young people to think critically about the news”—an initiative of the British weekly The Economist is such a step in the right direction, which should be copied by newspapers and TV, illustrating how they verify the sources of the news they report. Such programs should also be part of the curriculum at schools to fight the spread of unverified communications via social media.

Pandemic preparedness is thus not only a task for scientists and public health specialists, but also for sociologists and psychologists, as well as a broader public. We need discussions with and about the role and responsibilities of citizens in times of crisis. Notably, poets also have addressed pandemic preparedness in their writings, e.g. Albert Camus in “*La Peste*” (1947), where strict quarantine measures were taken. More recently, John Ironmonger in “*Not Forgetting the Whale”* (2017) described a fictious influenza pandemic seen from the perspective of a small village that gets self-organized. A marked solidarity develops spontaneously without governmental guidance. The story is a poetic allusion to Thomas Hobbes’ book *Leviathan* (1651), which expressed a pessimistic understanding of human nature. Before the state (and perhaps after the collapse of the state in a pandemic), man “lived in continual fear, and danger of violent death; and the life of man is solitary, poor, nasty, brutish and short.” Unfortunately, Hobbes’ conclusions on human societies lacking a state order and the rule of law are not far off the reality of failed states today (consider Sudan, Syria, Myanmar, and many other similar cases). Both Hobbes’ Leviathan and Ironmonger’s Whale are works of philosophical and poetic imagination. To settle what view better describes human behavior, we need sociological and psychological studies from the COVID-19 pandemic with data how societies, social groups, and individuals reacted during the pandemic.

Governments ordered the wearing of face masks, controlled vaccination certificates for entry to public places, closed schools, and imposed lockdowns. Part of the population accepted these measures for the common good of disease containment, while other parts of the population resisted these measures as a violation of their personal freedom. Did governments transgress their authority when ordering these restrictions? The conflict between individual rights and rules imposed by the governments for protecting the common good became very apparent in the COVID-19 pandemic. This conflict highlights that our societies must come to a consensus on what is the role of governments in general and in particularly during a crisis. What part of the sovereignty of an individual or of “We, the people” is in fact delegated in democratic systems to elected representatives in parliaments and governments who act for the common good (e.g. by raising taxes for building schools and hospitals and national defense)? The populist movements in the Western world question the role of governments and of elected representatives, and this is not only a problem for pandemic preparedness but for democratic order. It is important that a discussion on control measures and their legal basis is conducted before the next pandemic so that it can be done without undue emotional involvement. Climate and environmental change, ongoing growth of the world population, and economic and ecological migrations are conditions favoring a next pandemic. A close international collaboration coordinated by the WHO, consisting of a worldwide vaccination program and a tight surveillance and elimination of the last infection foci resulted in the past in the eradication of the smallpox virus. Vaccination efforts to eliminate measles virus and poliovirus should be intensified also to stimulate the feeling of human solidarity to a joint global task to create a spirit commensurate with a pandemic treaty.

When considering the impact of pandemics, it appears that pandemic preparedness and pandemic countermeasures cannot be handled at the level of individuals, but needs knowledge, financial capacity, and material resources of governments if not international organizations. Here enters a new element into the political discussion: The populists’ rejection of elites, including not only politicians but also of science. Scientists have a certain set of knowledge (of course continually updated or reversed by new evidence) that is essential to guide rational decisions. Scientists should not claim knowledge when, in a given situation and particularly in the beginning phase of an unfolding pandemic, this knowledge still does not exist or is fragmentary. Scientists should clearly formulate what is known at a given moment, what is not known, what is likely to be true, and what is likely or certainly false. The public also needs to be better instructed how scientific research is conducted in order to create trust in science.

Both the political and the scientific public discussions need mature citizens, open to arguments and also equipped with basic knowledge on politics and science. Our societies should look for more political and scientific education starting, but not ending in schools. Scientific organizations particularly in times of a crisis should also reach out to the public in a language that is broadly understandable. It becomes increasing clear that an anti-rationalism followed by some populist movements, fake news, and lies spread by malevolent groups or dictatorial governments via social media is a real danger not only for pandemic preparedness, but for free societies in general. History tells us that democracies lacking democrats as citizens are doomed to fail. Political education and Internet literacy are therefore important elements for a free society and the development of trust. Educating a mature and informed citizen is thus a critical element of all pandemic preparedness measures. The communication of basic knowledge on microbiology and infectious diseases to a broader public should therefore be an important task for learned societies in microbiology and infectious diseases. Adherence to the fair play of free societies founded on representative democratic order, the rule of law, the respect of the constitution, and tolerance to opposing opinions should likewise be tasks for societal groups ranging from political parties over churches to sports clubs, from families to friends to the work and school environment.

## Data Availability

This is a review article based on published and quoted literature. No new data were generated or analyzed in support of this research.

## References

[bib1] Abaluck J, Kwong LH, Styczynski A et al. Impact of community masking on COVID-19: a cluster-randomized trial in Bangladesh. Science. 2022;375:eabi9069. 10.1126/science.abi9069 34855513 PMC9036942

[bib2] Årdal C, Balasegaram M, Laxminarayan R et al. Antibiotic development—economic, regulatory and societal challenges. Nat Rev Micro. 2020;18:267–74. 10.1038/s41579-019-0293-3 31745330

[bib3] Baird LG, Banken R, Eichler HG et al. Accelerated access to innovative medicines for patients in need. Clin Pharmacol Ther. 2014;96:559–71. 10.1038/clpt.2014.145 25006877

[bib4] Balasegaram M, Piddock LJV. The Global Antibiotic Research and Development Partnership (GARDP) not-for-profit model of antibiotic development. ACS Infect Dis. 2020;6:1295–8. 10.1021/acsinfecdis.0c00101 32406675

[bib5] Beigel JH, Uyeki TM. SARS-Cov-2/COVID-19: clinical characteristics, prevention and treatment. In: Howley PM, Knipe DM (eds.), Field’s Virology Vol. 3 RNA Viruses. Philadelphia, PA: Wolters Kluwer, 2023, 741–84.

[bib6] Böhmer MM, Buchholz U, Corman VM et al. Investigation of a COVID-19 outbreak in Germany resulting from a single travel-associated primary case: a case series.Lancet Infect Dis. 2020;20:920–8. 10.1016/S1473-3099(20)30314-5 32422201 PMC7228725

[bib7] Brüssow H. Probiotics and prebiotics in clinical tests: an update. F1000Res. 2019;8:1157. Faculty Rev-1157. 10.12688/f1000research.19043.1 PMC6652097 31354938

[bib8] Brüssow H. Clinical trials with antiviral drugs against COVID-19: some progress and many shattered hopes. Environ Microbiol. 2021;23:6364–76. 10.1111/1462-2920.15769 34519154 PMC8652531

[bib9] Brüssow H. mRNA vaccines against COVID-19: a showcase for the importance of microbial biotechnology. Microb Biotechnol. 2022a;15:135–48. 10.1111/1751-7915.13974 34788497 PMC8652446

[bib10] Brüssow H. The beginning and ending of a respiratory viral pandemic-lessons from the Spanish flu. Microb Biotechnol. 2022b;15:1301–17. 10.1111/1751-7915.14053 35316560 PMC9049621

[bib11] Brüssow H. Viral infections at the animal–human interface: learning lessons from the SARS-CoV-2 pandemic. Microb Biotechnol. 2023;16:1397–411. 10.1111/1751-7915.14269 37338856 PMC10281366

[bib12] Brüssow H. Pandemic preparedness: on the efficacy of non-pharmaceutical interventions in COVID-19 and about approaches to predict future pandemic viruses. Microb Biotechnol. 2024a;17:e14431. 10.1111/1751-7915.14431 38465466 PMC10926049

[bib13] Brüssow H. Avian influenza virus cross-infections as test case for pandemic preparedness: from epidemiological hazard models to sequence-based early viral warning systems. Microb Biotechnol. 2024b;17:e14389. 10.1111/1751-7915.14389 38227348 PMC10832514

[bib14] Brüssow H. The antibiotic resistance crisis and the development of new antibiotics. Microb Biotechnol. 2024c;17:e14510. in press. 10.1111/1751-7915.14510 38970161 PMC11226406

[bib15] Brüssow H, Brüssow L. Clinical evidence that the pandemic from 1889 to 1891 commonly called the Russian flu might have been an earlier coronavirus pandemic. Microb Biotechnol. 2021;14:1860–70. 10.1111/1751-7915.13889 34254725 PMC8441924

[bib16] Carlson CJ, Albery GF, Merow C et al. Climate change increases cross-species viral transmission risk. Nature. 2022;607:555–62. 10.1038/s41586-022-04788-w 35483403

[bib17] Cassini A, Högberg LD, Plachouras D et al. Attributable deaths and disability-adjusted life-years caused by infections with antibiotic-resistant bacteria in the EU and the European Economic Area in 2015: a population-level modelling analysis. Lancet Infect Dis. 2019;19:56–66. 10.1016/S1473-3099(18)30605-4 30409683 PMC6300481

[bib18] Chan PF. Finding the gems using genomic discovery: antibacterial drug discovery strategies—the successes and the challenges. Drug Discov Today: Ther Strategies. 2004;1:519–27.

[bib19] Chen YM, Hu SJ, Lin XD et al. Host traits shape virome composition and virus transmission in wild small mammals. Cell. 2023;186:4662–4675.e12. 10.1016/j.cell.2023.08.029 37734372

[bib20] Cohen J. Global pandemic treaty stalls, again, over equity concerns. Science. 2024a; 384:1054 10.1126/science.adq8854 38843344

[bib21] Cohen J. Worries about bird flu in U.S. cattle intensify. Science. 2024b;384:12–13. 10.1126/science.adp6024 38574129

[bib22] Cook MA, Wright GD. The past, present, and future of antibiotics. Sci Transl Med. 2022;14:eabo7793. 10.1126/scitranslmed.abo7793 35947678

[bib23] Cutler DM, Summers LH. The COVID-19 pandemic and the $16 trillion virus. JAMA. 2020;324:1495–6. 10.1001/jama.2020.19759 33044484 PMC7604733

[bib24] Darrow JJ, Avorn J, Kesselheim AS. FDA approval and regulation of pharmaceuticals, 1983–2018. JAMA. 2020;323:164–76. 10.1001/jama.2019.20288 31935033

[bib25] de Kraker ME, Stewardson AJ, Harbarth S. Will 10 million people die a year due to antimicrobial resistance by 2050?. PLoS Med. 2016;13:e1002184. 10.1371/journal.pmed.1002184 27898664 PMC5127510

[bib26] Dheman N, Mahoney N, Cox EM et al. An analysis of antibacterial drug development trends in the United States, 1980–2019. Clin Infect Dis. 2021;73:e4444–50. 10.1093/cid/ciaa859 32584952

[bib27] Else H. Tallying the health toll of COVID-19. Nature. 2022;605:410–3. 10.1038/d41586-022-01341-7 35585337

[bib28] Engel A. Fostering antibiotic development through impact funding. ACS Infect Dis. 2020;6:1311–2. 10.1021/acsinfecdis.0c00069 32527099

[bib30] GBD 2019 Diseases and Injuries Collaborators. Global burden of 369 diseases and injuries in 204 countries and territories, 1990–2019: a systematic analysis for the Global Burden of Disease Study 2019. Lancet North Am Ed. 2020;396:1204–22. 10.1016/S0140-6736(20)30925-9 PMC7567026 33069326

[bib31] GBD 2019 Diseases and Injuries Collaborators. Global mortality associated with 33 bacterial pathogens in 2019: a systematic analysis for the Global Burden of Disease Study 2019. Lancet North Am Ed. 2022;400:2221–48. 10.1016/S0140-6736(22)02185-7 PMC9763654 36423648

[bib33] He WT, Hou X, Zhao J et al. Virome characterization of game animals in China reveals a spectrum of emerging pathogens. Cell. 2022;185:1117–1129.e8. 10.1016/j.cell.2022.02.014 35298912 PMC9942426

[bib34] Hsia Y, Lee BR, Versporten A et al. Use of the WHO Access, Watch, and Reserve classification to define patterns of hospital antibiotic use (AWaRe): an analysis of paediatric survey data from 56 countries. Lancet Glob Health. 2019;7:e861–71. 10.1016/S2214-109X(19)30071-3 31200888

[bib35] Karthikeyan S, Levy JI, De Hoff P et al. Wastewater sequencing reveals early cryptic SARS-CoV-2 variant transmission. Nature. 2022;609:101–8. 10.1038/s41586-022-05049-6 35798029 PMC9433318

[bib36] Last JM (ed). A Dictionary of Epidemiology, 4th edn. New York: Oxford University Press, 2001.

[bib37] Lee B, Broder CC, Wang L-F. Henipaviruses: Hendra and Nipah viruses. In: Howley PM, Knipe DM (eds.), Field’s Virology Vol. 1 Emerging Viruses. Philadelphia, PA: Wolters Kluwer, 2021, 559–95.

[bib38] Leitner L, Ujmajuridze A, Chanishvili N et al. Intravesical bacteriophages for treating urinary tract infections in patients undergoing transurethral resection of the prostate: a randomised, placebo-controlled, double-blind clinical trial. Lancet Infect Dis. 2021;21:427–36. 10.1016/S1473-3099(20)30330-3 32949500

[bib39] Lewis K. The science of antibiotic discovery. Cell. 2020;181:29–45. 10.1016/j.cell.2020.02.056 32197064

[bib40] McKenna M. What a treatment for “super gonorrhoea” means for future drug development. Nature. 2024;626:942–4. 10.1038/d41586-024-00543-5 38418910

[bib41] Mitze T, Kosfeld R, Rode J et al. Face masks considerably reduce COVID-19 cases in Germany. Proc Natl Acad Sci USA. 2020;117:32293–301. 10.1073/pnas.2015954117 33273115 PMC7768737

[bib42] Muñoz KA, Ulrich RJ, Vasan AK et al. A Gram-negative-selective antibiotic that spares the gut microbiome. Nature. 2024;630:429–36. 10.1038/s41586-024-07502-0 38811738 PMC12135874

[bib43] Outterson K, Rex JH. Global pull incentives for better antibacterials: the UK leads the way. Appl Health Econ Health Policy. 2023;21:361–4. 10.1007/s40258-023-00793-w 36773115 PMC10119039

[bib44] Perlman S, Masters PS. Coronaviridae: the viruses and their replication. In: Howley PM, Knipe DM (eds.), Field’s Virology Vol. 1 Emerging Viruses. Philadelphia, PA: Wolters Kluwer, 2021, 410–48.

[bib45] Pirnay JP, Djebara S, Steurs G et al. Personalized bacteriophage therapy outcomes for 100 consecutive cases: a multicentre, multinational, retrospective observational study. Nat Microbiol. 2024;9:1434–53. 10.1038/s41564-024-01705-x 38834776 PMC11153159

[bib46] Rex JH, Outterson K. Antibacterial R&D at a crossroads: we've pushed as hard as we can … now we need to start pulling!. Clin Infect Dis. 2021;73:e4451–3. 32584949 10.1093/cid/ciaa852

[bib47] Santos-Júnior CD, Torres MDT, Duan Y et al. Discovery of antimicrobial peptides in the global microbiome with machine learning. Cell. 2024;187:3761–3778.e16. 10.1016/j.cell.2024.05.013 38843834 PMC11666328

[bib48] Sarker SA, Sultana S, Reuteler G et al. Oral phage therapy of acute bacterial diarrhea with two coliphage preparations: a randomized trial in children from Bangladesh. EBioMedicine. 2016;4:124–37. 10.1016/j.ebiom.2015.12.023 26981577 PMC4776075

[bib49] Schlander M, Hernandez-Villafuerte K, Cheng CY et al. How much does it cost to research and develop a new drug? A systematic review and assessment. Pharmacoeconomics. 2021;39:1243–69. 10.1007/s40273-021-01065-y 34368939 PMC8516790

[bib50] Scully EP, Kuritzkes DR. HIV-1: pathogenesis, clinical manifestations, and treatment. In: Howley PM, Knipe DM (eds.), Field’s Virology Vol. 3 RNA Viruses. Philadelphia, PA: Wolters Kluwer, 2023, 618–47.

[bib51] Taylor SN, Marrazzo J, Batteiger BE et al. Single-dose zoliflodacin (ETX0914) for treatment of urogenital gonorrhea. N Engl J Med. 2018;379:1835–45. 10.1056/NEJMoa1706988 30403954

[bib52] Thadani NN, Gurev S, Notin P et al. Learning from prepandemic data to forecast viral escape. Nature. 2023;622:818–25. 10.1038/s41586-023-06617-0 37821700 PMC10599991

[bib53] Timmis K, Brüssow H. The COVID-19 pandemic: some lessons learned about crisis preparedness and management, and the need for international benchmarking to reduce deficits. Environ Microbiol. 2020;22:1986–96. 10.1111/1462-2920.15029 32319151 PMC7264722

[bib54] Tommasi R, Brown DG, Walkup GK et al. ESKAPEing the labyrinth of antibacterial discovery. Nat Rev Drug Discov. 2015;14:529–42. 10.1038/nrd4572 26139286

[bib55] Uyttebroek S, Chen B, Onsea J et al. Safety and efficacy of phage therapy in difficult-to-treat infections: a systematic review. Lancet Infect Dis. 2022;22:e208–20. 10.1016/S1473-3099(21)00612-5 35248167

[bib56] van den Akker CHP, van Goudoever JB, Shamir R et al. Probiotics and preterm infants: a position paper by the European Society for Paediatric Gastroenterology Hepatology and Nutrition Committee on Nutrition and the European Society for Paediatric Gastroenterology Hepatology and Nutrition Working Group for Probiotics and Prebiotics. J Pediatr Gastroenterol Nutr. 2020;70:664–80. 32332478 10.1097/MPG.0000000000002655

[bib57] Wouters OJ, McKee M, Luyten J. Estimated research and development investment needed to bring a new medicine to market, 2009–2018. JAMA. 2020;323:844–53. 10.1001/jama.2020.1166 32125404 PMC7054832

[bib58] Ye T, Jiao Z, Li X et al. Inhaled SARS-CoV-2 vaccine for single-dose dry powder aerosol immunization. Nature. 2023;624:630–8. 10.1038/s41586-023-06809-8 38093012

[bib59] Zanichelli V, Sharland M, Cappello B et al. The WHO AWaRe (Access, Watch, Reserve) antibiotic book and prevention of antimicrobial resistance. Bull World Health Org. 2023;101:290–6. 10.2471/BLT.22.288614

[bib60] Zhang XA, Li H, Jiang FC et al. A zoonotic henipavirus in febrile patients in China. N Engl J Med. 2022;387:470–2. 10.1056/NEJMc2202705 35921459

